# The Primary Mental Health Care Project: an example of triple integration

**DOI:** 10.3399/bjgpopen17X100797

**Published:** 2017-04-19

**Authors:** Catie Nagel, Kristin Bash

**Affiliations:** 1 GP Partner and Clinical Teaching Fellow University of Leeds, Academic Unit of Public Health & Primary Care, Leeds Institute of Health Sciences, University of Leeds, Leeds, UK; 2 Public Health Specialty Registrar, Sheffield City Council, Sheffield, UK

**Keywords:** mental health, integration, social care

## Introduction

Despite the publication of a number of governmental policy documents asserting the need for parity of esteem between mental and physical health care,^1,2^ it remains the case that patients with severe mental illness often receive a service that is inadequate to meet their needs or improve outcomes.^3,4^ The costs to individuals and society are large, with over £11 billion spent by the NHS in England annually on functional illness and comorbidity arising from mental and physical health problems^5^ and an estimated total societal cost of schizophrenia in England of £6.7 billion.^6^ The need to integrate physical and mental health care was highlighted in the NHS's *Five Year Forward View* and was subsequently described as the ‘new frontier for integrated care’ by the King’s Fund.^5,7^ The term ‘triple integration’ was used by Simon Stevens to describe new models of care proposed in the *Five Year Forward View*; models in which integration of primary and specialist care, physical and mental health, and health and social care occurred.^7^ This article presents a case study that offers a model of integrated care.

Pitsmoor Surgery serves a population with twice the city average and three times the national average of severe enduring mental illness (SEMI). The Primary Mental Health Care Project (PMHCP) was set up by one of the project partners in the mid-1990s, as it was recognised that the needs of this population were being insufficiently met by the services available at the time. The project provides a holistic model of care to patients aged >16 years, suffering with a variety of enduring mental health problems, from severe depression and anxiety to schizophrenia, personality disorder, and bipolar disorder. The project aims to promote psychological, social, and physical wellbeing among its clients. The surgery employs and manages the project staff directly and is part-funded by the Sheffield Clinical Commissioning Group (CCG), clinically-led statutory NHS bodies responsible for planning and commissioning health services in their local area. The team is small, but skilled, with one worker hailing from a social work background and one from nursing. Two out of the three workers have formal psychotherapy qualifications. Referrals from neighbouring practices are welcomed and the project also offers training, consultancy, and external supervision to other local organisations working with patients suffering SEMI. Historically, project workers have been able to access the remaining funding needed to run the project from a variety of sources: top-up funding from grant making trusts, charitable organisations, or so-called ‘freed up resources’ from the CCG. The current financial climate has meant that finding this additional funding has become an increasing challenge.

Support to patients and their carers is offered through individual one-to-one sessions, as well as through group and social activities such as the ‘knit and natter’ group and the healthy walks scheme. The project has given rise to a successful offshoot organisation offering horticultural therapy. Some interventions are short-term, but other clients are seen over many years, where a Care Programme Approach^8^ is taken. The retention rate of staff has been excellent and all members of staff have been working with the project from between 10 and 20 years. This continuity has been particularly important for the patient group the project serves.

## Does it work?

A service provision review was commissioned by the Sheffield CCG and undertaken early in 2016. Semi-structured interviews took place with PMHCP staff, a neighbouring GP comparator practice (not served by the project), staff from a local community mental health team (CMHT), and a provider and commissioner from a third sector community support organisation. The CMHT provides secondary care mental health services. Three main themes emerged following analysis of seven semi-structured interviews with the above listed participants. An iterative, Grounded Theory approach^9^ was taken with the emergent themes from each interview informing subsequent topic guides.

## Findings

Three themes were identified following the analysis of transcribed data. These included:

- Challenges involved in supporting patients who are reluctant to engage with services
- Patient-centred, holistic care and ‘handholding’
- Importance of trust and relationship building between support worker and patient

### Reluctance to engage

The majority of patients cared for by the PMHCP had either been discharged from secondary care services or had never managed to successfully engage with this service. There were a number of factors relating to why these patients were not under formal psychiatric follow-up, relating to access, thresholds for care, and discharge from services following a period of poor engagement. Some patients had cited negative feelings towards the psychiatric services following previous difficult experiences (such as ‘sectioning’ under the Mental Health Act) and the patient group were generally more trusting of community-based services and the GP.

### Patient-centred care and ‘handholding’

A participant from the CMHT highlighted the fact that they had limited resources to follow-up patients that were engaging poorly with their services. Those patients who were not deemed to pose a significant risk to themselves or others at the time of assessment, were frequently discharged following a period of non-engagement. The interviewed participant described aspects of the service provided by the PMHCP as being akin to their ‘assertive outreach team,’ as the project staff recognised the need for flexibility and increased allocation of resources to those more anxious or difficult to access patients. ‘Handholding’ frequently took place to get patients to a first meeting with a new GP, a weight management service, or a third sector organisation, such as the Citizens Advice Bureau. This has not only helped bring these patients into contact with the most appropriate agency, but has also helped us to provide better physical health care, with increased support for patients to attend and engage in screening and recalls for immunisations and health checks.

### Trust and relationships

Continuity of care was identified as a factor that made this model highly effective. The retention of staff to the project has been a measure of its success and this continuity has helped to foster positive relationships between patients and professionals, as well as enhanced working relationships between the project and practice staff and secondary care colleagues. An environment of trust and openness between project staff and the host practice was cited as an important facilitator of good care, as this has helped us to develop a common set of values and working practices.

Fragmentation of care was identified as an important issue by the participant from the comparator practice. Breakdown of trust and relationships was noted to be a key factor in poor engagement, particularly when patients and their carers were unsure who to turn to in times of crisis.

Close working relationships between project and practice staff allowed for higher risk tolerance and staff felt more able to hold and manage risk, since they were well supported by the GPs in the host practice. PMHCP staff have well-established links with secondary care colleagues at the CMHT, which has further enabled them to tolerate risk and reduce re-referral to specialist services.

## Benefits

The PMHCP offers a holistic model of care for patients with serious and enduring mental illness who are currently not under active follow-up from the CMHT. It cares for those whose needs would not be well met by either the NHS's Improving Access to Psychological Therapies (IAPT) programme, or the third sector organisations currently available, because the needs of these patients are too complex and longstanding. Integration of the project within the practice environment has been a strength and has helped enable the project to provide better physical health care for this group of patients. Social aspects of care are considered alongside mental health needs by project staff and practical input is offered to help patients access the most appropriate service to meet their needs and to ameliorate social isolation. While the project has not been profit generating for this practice, it is possible that with a scaled-up model of activity, inclusive of more paid activity, such as involvement in training for external organisations, the project could be more sustainable and even provide additional income for the host practice.

## Challenges

The operational model of the PMHCP has been its strength and its weakness. By offering a way of working that is fluid, holistic, and multifaceted, it is better able to meet patient needs, but this makes communicating the remit and outputs of the project more challenging. As with any intervention, the question of how to measure success must be asked and in the challenging financial climate, the need to ascertain value for money cannot be ignored.

The project is currently part-funded and additional funding needs to be sought on an annual basis or absorbed by the practice, in order for it to remain viable. Additional grants have been challenging to obtain, resulting in the need to scale down the working hours of project staff. Time to seek additional funding streams is therefore increasingly limited, and the problem of sustainability risks becoming self-perpetuating. This model of care has worked well and successfully for over 20 years, albeit at a small scale, but with increasing pressure on budgets, it remains to be seen whether or not it continues to be sustainable.

Given such pressures, practices wishing to employ a similar model of care are advised to consider how outcomes can be demonstrated from the outset. Valid assessment tools are now in place to help facilitate such work and the use of the Recovering Quality of Life (ReQoL)^10^ questionnaire has since been implemented, with the aim of assessing the impacts of the PMHCP on the quality of life of the patients it serves.

As previously mentioned, the authors feel that the PMHCP is an example of what Stevens referred to as triple integration.^5^ Now more than ever, such a model is needed to help meet the needs of this underserved and vulnerable population. The authors hope that they can continue to build on its success.

## Abbreviations and Glossary

IAPT: Improving Access to Psychological Therapies. An NHS programme which offers a variety of short term interventions including Cognitive Behavioural Therapy or Counselling.
CCG: Clinical Commissioning Group. Clinically led statutory NHS bodies responsible for planning and commissioning health care services in their local area.
CAB: Citizens Advice Bureau. An advisory service for people needing help or advice with relation to matters including benefits, housing, or personal debt.
CMHT: Community Mental Health Team. A secondary care provider of mental health services.
